# Shotgun proteomic analysis of S-thiolation sites of guinea pig lens nuclear crystallins following oxidative stress in vivo

**Published:** 2013-02-03

**Authors:** Frank J. Giblin, Larry L. David, Phillip A. Wilmarth, Victor R. Leverenz, M. Francis Simpanya

**Affiliations:** 1 Eye Research Institute, Oakland University, Rochester, MI; 2 Biochemistry and Molecular Biology, Oregon Health & Science University, Portland, OR

## Abstract

**Purpose:**

To compare levels of S-glutathiolation and S-cysteinylation occurring at more than 60 cysteine residues of 12 different guinea pig lens water-soluble nuclear crystallins following treatment of the animals with hyperbaric oxygen (HBO).

**Methods:**

Guinea pigs (initially 18 months old) were treated 30X (3X per week for 10 weeks) with HBO (2.5 atm 100% O_2_ for 2.5 h) as a model to study the formation of nuclear cataract. This treatment produces a moderate increase in lens nuclear light scatter (compared to denser scatter occurring after 80 HBO treatments), with five- to sixfold increases in levels of protein-bound glutathione (PSSG) and protein-bound cysteine (PSSC). Trypsin digests of lens nuclear water-soluble proteins were analyzed with two-dimensional liquid chromatography and mass spectrometry to identify specific cysteine residues binding either glutathione or cysteine. Lens nuclei of age-matched untreated animals were used as controls.

**Results:**

All major crystallins, except αB, were modified to some extent by either S-glutathiolation or S-cysteinylation. Overall, 72% of the cysteine residues of guinea pig lens nuclear crystallins were shown to be capable of binding glutathione, cysteine, or both molecules. The crystallin with the highest level of modification was βA1/A3 (six of eight –SH groups), and that with the lowest (two of five –SH groups) was βA2. O_2_-induced increases in PSSG levels were 2.8, 2.4, and 4.1 times control for γA-, γB-, and γC-crystallins, respectively. Comparable increases in PSSC levels for the three γ-crystallins were 2.3, 2.7, and 2.4 times control, respectively. βB2-crystallin showed the highest amount of O_2_-induced PSSG formation of any of the crystallins, as well as a substantial level of control PSSG, and nearly all of this was due to a single residue, C67, a site also present in human βB2-crystallin. Overall, 32 of the 44 modified cysteine residues were homologous with the human.

**Conclusions:**

This large-scale study successfully identified lens crystallin cysteine residues that bound glutathione and/or cysteine under normal or oxidative stress conditions. The high percentage of protein –SH groups that are modified by S-thiolation in the guinea pig lens nucleus demonstrates the substantial protein sulfhydryl redox buffer capability present in the center of the lens. The results suggest that PSSG and PSSC formation may act to delay O_2_-induced insolubilization of γA-, γB-, and γC-crystallins, and β-crystallins, but with a greater effect on the γ-crystallins at an early stage of oxidative stress. The study has shown that technological approaches are now available to investigate in considerable detail the role of specific lens –SH groups in nuclear cataractogenesis.

## Introduction

The mammalian lens contains an unusually high concentration of protein –SH (PSH) groups, approaching 50 mM in the central nuclear region [1-3]. The approximately one-dozen crystallin proteins in the lens possess more than 60 different cysteine residues. The γ-crystallins, for example, contain seven or more –SH groups per molecule, while αB is the only crystallin that has none. Why such a high level of potentially oxidizable residues exists in the center of the mammalian lens remains an unanswered question in lens and cataract research [4] (in contrast, the nucleus of the bird lens is devoid of PSH [2]). Evidence indicates that high levels of soluble PSH can function as an active cellular redox buffer to protect against oxidative stress [5]. Human nuclear cataract, one of the most common types [6,7], is closely associated with oxidative stress and oxidation of cysteines [8]. Mature forms of nuclear cataract can have more than 90% of crystallin –SH groups oxidized to protein disulfide (PSSP) [9,10]. In addition, it has been reported that once human nuclear cataracts begin to develop, they progress rapidly [11], possibly an indication of the potential instability of crystallin –SH groups in the lens center.

In addition to formation of PSSP in the aging human lens and in nuclear cataracts, there is also an increase in the levels of protein-thiol mixed disulfides, occurring via the process of protein S-thiolation [12]. Mixed disulfides in the lens include protein-bound glutathione (PSSG) formed by S-glutathiolation and protein-bound cysteine (PSSC) formed by S-cysteinylation [13,14]. As a possible precursor to the formation of PSSP, protein S-thiolation is a common and rapid reaction to oxidative stress [15,16]. The formation of mixed disulfides may be a homeostatic protective mechanism, acting to delay the oxidation of crystallin –SH groups to irreversible disulfide forms [17]. Analysis of disulfide in human lens water-insoluble protein has shown the presence of PSSP only, with no PSSG [18].

Molecular oxygen (O_2_) has been implicated in the formation of maturity-onset nuclear cataract [19-21]. It has been hypothesized that age-related liquefaction of vitreous humor and formation of posterior vitreous detachment may allow O_2_ to travel from the retinal vasculature to the lens nucleus, causing oxidation-induced loss of transparency in that region [22,23]. Therapeutic treatment of patients with hyperbaric oxygen (HBO) for long periods can lead to the development of nuclear cataract [24]. We have developed a model for studying the early stages of nuclear cataract by treating guinea pigs with HBO [1,25], which produces increased levels of lens nuclear light scattering, as well as elevated levels of PSSP in the lens nucleus, but not in the cortex [26]. One of the earliest indicators of oxidative stress in our guinea pig/HBO model is an increase in PSSG and PSSC levels in the lens nucleus [1].

Little is known about the relative susceptibility of specific crystallin –SH groups in the lens nucleus to form PSSG and PSSC during oxidative stress. In the present study, we used mass spectrometry to investigate formation of PSSG and PSSC at specific water-soluble crystallin sites in the lens nucleus of guinea pigs treated with HBO. We employed strong cation exchange (SCX), reverse phase liquid chromatography, and tandem mass spectrometry (MS/MS) to separate and identify enzyme-digested peptides [27]. In this study, we used 30 HBO treatments of guinea pigs over a 2.5-month period since this was previously shown to produce peak levels of PSSG and PSSC in the lens nucleus of the animals [1]. When guinea pigs are treated more than 30 times with HBO, there is a shift to formation of PSSP in the lens nucleus, with increased precipitation of crystallins. The results of the current study demonstrated the wide variety of specific crystallin –SH groups in the lens nucleus able to bind glutathione, cysteine, or both molecules, and the considerable PSH redox buffering capacity existing in the guinea pig lens nucleus to minimize irreversible PSSP formation.

## Methods

### Animals

All animal care and other work performed in the study conformed to the Association for Research in Vision and Ophthalmology statement for the use of animals in ophthalmic and vision research, and the U.S. Department of Agriculture standards. Male retired breeder Hartley guinea pigs, initially 17 to 18 months old, were obtained from Kuiper Rabbit Ranch (Indianapolis, IN). The animals were held for 1 to 2 weeks before HBO treatment, to allow recovery from the stress of shipment and to identify the healthiest animals for the study. During this period, the lenses of the guinea pigs were examined carefully with slit-lamp biomicroscopy, and animals with cortical or nuclear opacities were excluded.

### Hyperbaric oxygen treatment

Details of HBO treatment of guinea pigs have been previously reported [26]. Briefly, the animals were treated three times per week, on alternate days, with 2.5 atm of 100% O_2_, for 2.5 h periods. Thirty treatments were administered over a 2.5-month period. The effect of HBO treatment on lens nuclear light scattering was assessed with a slit-lamp microscope. Euthanization of one guinea pig at a time was conducted using a Euthanex Auto CO_2_ System (E-Z Systems, Inc., Palmer, PA). The animal was exposed to CO_2_ for 5 min at a flow rate that varied from 1.75 to 2.4 ft^3^/min. Eyes were enucleated, and the lenses removed by posterior approach and placed immediately on dry ice. The lenses were stored in liquid nitrogen until analysis.

### Preparation of protein samples

Lenses from the control and HBO-treated animals were dissected into the nucleus and cortex with use of a cork borer [26]. Only the lens nuclei, comprising 25% of the lens weight, were used for this study. The nuclei were homogenized in a 10×volume of 20 mM sodium phosphate buffer (pH 7.0), containing 1 mM EDTA and 50 mM iodoacetamide under a nitrogen atmosphere to prevent artifactual oxidation of the –SH groups. Two guinea pig lens nuclei, with a total wet weight of 40 mg, were sufficient for completing the mass spectrometry analysis. The homogenate was centrifuged for 25 min at 15,000 ×g at 4 °C to isolate water-soluble (WS) proteins. Water-insoluble proteins were not analyzed in this study. Protein concentrations were measured using BCA Protein Assay reagent (Pierce, Rockford, IL) with bovine serum albumin as a standard. WS proteins were divided into aliquots of 2 mg protein and freeze-dried.

### Protein digestion

Two mg of freeze-dried protein of HBO-treated and age-matched control lens nuclei were each reconstituted in 200 µl of 1 M Tris-HCl digestion buffer, pH 8.5, containing 8 M urea, 200 mM methylamine, and 8 mM CaCl_2_. The samples were diluted with water and 80 µg of trypsin Gold (Promega Corporation, Madison, WI) was added to each 2 mg protein sample (i.e., 1 µg of trypsin to 25 µg of protein) so that the final volume was 800 µl. A disulfide bond–reducing agent was not employed to be able to investigate modification of crystallin cysteine residues by S-glutathiolation and S-cysteinylation. An aliquot of the protein sample was taken before and after overnight trypsin digestion at 37 °C to check completion of the protein digest using sodium dodecyl sulfate–polyacrylamide gel electrophoresis. Protein digest peptides were desalted using Sep-Pak cartridges (Waters Corporation, Milford, MA).

### Strong cation- exchange high-performance liquid chromatography

Peptides were injected onto a polysulfoethyl A cation exchange column (100×2.1 mm; Nest Group, Inc., Southborough, MA). The flow rate for the column was 200 µl/min. The composition of buffer A was 10 mM sodium phosphate (pH 3.0) containing 25% acetonitrile. Buffer B had the same composition as buffer A, except for the addition of 350 mM KCl. The peptides were loaded onto the SCX column, washed using buffer A for 15 min, and then eluted using a gradient of 0%–50% buffer B for 45 min and 50%–100% buffer B for 20 min. Fractions were collected at 60-s intervals and pooled into 29 fractions based on the relative ultraviolet (UV) absorbance of each fraction at 280 nm. The pooled fractions were then dried and reconstituted in 100 µl of 5% formic acid.

### Liquid chromatography/mass spectrometry and data acquisition

Twenty-percent portions of the reconstituted SCX fractions were separated using reverse-phase chromatography with an Agilent 1100 series capillary liquid chromatography (LC) system (Agilent Technologies, Palo Alto, CA), and the peptides were analyzed using a linear trap quadrupole (LTQ) linear ion trap mass spectrometer with an Ion Max electrospray ionization source fitted with a 34-gauge metal needle (Thermo Scientific, San Jose, CA). Electrospray was performed without sheath gas at 2.7 kV potential. Xcalibur (Thermo Scientific, version 2.07 SP1) was used to control the system. Samples were applied at 20 µl/min to a trap cartridge (Michrom BioResources, Auburn, CA), and then switched onto a 0.5×250 mm Zorbax SB-C18 column with 5 μm particles (Agilent Technologies) using a mobile phase containing 0.1% formic acid, a 7%–30% acetonitrile gradient over 100 min, and a 10 µl/min flow rate. Collision-induced dissociation used a normalized collision energy of 35%. Data-dependent collection of MS/MS spectra used dynamic exclusion (repeat count equal to 1, exclusion list size of 50, exclusion duration of 30 s, and exclusion mass width of −1 to +4 Da) to obtain MS/MS spectra of the three most abundant parent ions (minimum signal of 5,000) following each survey scan from m/z 400–2,000. The tune file was configured with no averaging of microscans, a maximum inject time of 200 msec, and automatic gain control targets of 3×10^4^ in the MS1 mode and 1×10^4^ in the MS2 mode.

### Mass spectrometry data analysis

#### Database

Version 62 of the Ensembl FASTA protein database (19,774 *Cavia porcellus* sequences) was downloaded on June 1, 2011. We used a sequence-reversed database to estimate error thresholds [28]. The database sequences were appended with 179 common contaminant sequences, and reversed forms for all sequences were concatenated for a final database of 39,906 sequences. The database processing was performed with Python scripts available at ProteomicAnalysisWorkbench.

#### DTA creation

RAW data from the mass spectrometer were converted to DTA files representing individual MS2 spectra using DTA Extract in BioWorks (version 3.3; Thermo Scientific); charge state analysis was performed using the ZSA option in BioWorks. The group scan minimum count was 1, a minimum of 25 ions was required, the mass tolerance for combining DTAs was set to prevent combining DTA files, and an absolute intensity threshold of 500 was used.

#### Lens proteome determination

SEQUEST (version 28, revision 12, Thermo Scientific) searches for all samples were performed with trypsin specificity, a maximum of two missed cleavages, an average parent ion mass tolerance of 2.5 Da, and a monoisotopic fragment ion mass tolerance of 1.0 Da. A static modification of +57 Da was added to all cysteine residues. We computed a linear discriminant transformation of SEQUEST scores [29,30] and created discriminant score histograms for each peptide charge state (1+, 2+, and 3+). Separate histograms were created for forward and reversed sequence matches for peptides of seven amino acids or longer. Reversed matches were used to estimate peptide false discovery rates (FDRs) and set score thresholds for each charge state to achieve a 1% peptide FDR. The sets of confidently identified peptides for each lens sample were collectively mapped to the protein database. Any proteins identified by identical sets of peptides were grouped together as redundant proteins. Any proteins identified by a peptide set that was a formal subset of another protein’s peptide set were removed (parsimony principle). Any proteins that were not identified by at least two distinct peptides having two tryptic termini per sample were removed, resulting in a final list of 520 confidently identified lens proteins (1% protein FDR).

#### Modified peptide detection

The lens proteome database (520 sequences and their reversed forms) was used in SEQUEST searches configured for no enzyme cleavage specificity, and with several variable modifications. The variable modifications were cysteine residues with an additional mass of 248 (the net mass of glutathione adducts in excess of the static cysteine alkylation mass of 57 Da), cysteine residues with an additional mass of 62 (net mass increase of cysteinylation given a static C+57 alkylation mass), and methionine with a mass increase of 16 Da. Score histograms were created for each charge state (1+, 2+, or 3+), for each number of tryptic termini (2, 1, or 0), and for each homogeneously modified peptide form having at most two modifications per peptide. Score thresholds were set at a 1% peptide FDR independently across the 36 score histograms. Any peptide classes with score histograms that lacked fewer than 20 target peptide match scores in excess of the highest-scoring decoy matches were excluded. Modified peptide score histograms for 2+ peptides are shown in the Appendix 1, and MS/MS spectra for modified peptides are shown in Appendix 2.

The small 1,040 protein database was necessary given the many-fold increase in search times due to nonspecific enzymatic cleavage and several variable modifications; however, using the small database significantly increased the chance that incorrectly identified peptides might match the 520 target lens proteins. Even with a strict 1% peptide FDR, the large data sets in this experiment resulted in large enough numbers of incorrect peptides that we used three distinct peptides per protein during results reporting to reduce the number of incorrect matches. Complete protein, peptide, and modified peptide results are tabulated in Appendix 3, Appendix 4 and Appendix 5. Protein FDR is not applicable in searches using databases of identified proteins, and was not computed.

#### Extent of cysteine oxidation

Spectral counting of modified peptides was used to probe the extent of cysteine oxidation/modification similar to our previous large-scale modification studies [31]. We normalized modified cysteine-containing peptide counts by observed total cysteine-containing peptide counts to control for any possible changes in soluble protein composition introduced by HBO treatment, sample-loading variation, and instrumental variation.

## Results

Global post-translational modification mapping is more feasible in the lens due to the high abundances of the major crystallins. Spectral counts for the taxon-specific ζ-crystallin made up nearly 16% of the total peptide counts and were about 1.6 times higher than those for the next highest crystallins, γS, βB2, αA, and γB (Table 1, columns 2 and 3). Counts for γB-crystallin (9.4% of the total) were higher than γC-crystallin, and nearly 3 times higher than γA-crystallin. αA-crystallin (9.7% of the total) had about twice as many total counts as αB. Detecting cysteine-containing peptides in the lower abundance crystallins, such as βA2, was more difficult. γN-crystallin had very low total counts, <0.2% of the total, and was not included in Table 1.

**Table 1:** Numbers of total crystallin spectral counts (an indication of relative crystallin abundance), cysteine-containing peptide spectral counts, number of –SH groups for each crystallin, and numbers of –SH groups bound by GSH or cysteine for each guinea pig lens water-soluble nuclear crystallin.

Crystallin*	Total spectral counts**	peptide counts as % of total	Cysteine-containing peptide counts**	Cysteine-peptide counts as % of total	Number of -SH groups per crystallin	-SH groups bound by GSH or cysteine***
ζ	4500	15.9	828	10.4	5	4
γS	2947	10.4	695	8.7	7	5
βB2	2794	9.9	516	6.4	2	2
αA	2722	9.7	510	6.4	1	1
γB	2664	9.4	1240	15.5	7	5
βB1	2299	8.2	865	10.8	5	4
γC	2226	7.9	680	8.5	7	5
βA4	1874	6.6	479	6	4	4
βA1/A3	1757	6.2	1173	14.7	8	6
αB	1597	5.7	0	0	0	0
βB3	1306	4.6	159	2	3	2
γA	922	3.3	709	8.9	7	4
βA2	613	2.2	140	1.7	5	2
total	28221	100	7994	100	61	44

* γN-crystallin was not included since it had very low numbers (<50) of MS/MS spectral counts. **Averages of control and hyperbaric oxygen-treated results. *** The number of –SH groups in either control or hyperbaric oxygen-treated samples that were found to bind either glutathione or cysteine.

Spectral counts for peptides containing an –SH group are also listed in Table 1 (column 4), along with –SH peptide counts as a percent of total counts for each crystallin (column 5). The –SH peptide counts were highest (>1000) for the γB- and βA1/A3-crystallins, each of which made up nearly 15% of the total. Counts were relatively low for the lower abundance βB3- and βA2-crystallins, each comprising 2% of the total (column 5). The highest number of –SH groups per crystallin, eight, was shown by βA1/A3-crystallin, followed by seven –SH groups each for the four γ-crystallins (column 6). Modified peptides were detected for 44 of the total 61 cysteine sites for the 12 crystallins (column 7).

**Figure 1:**
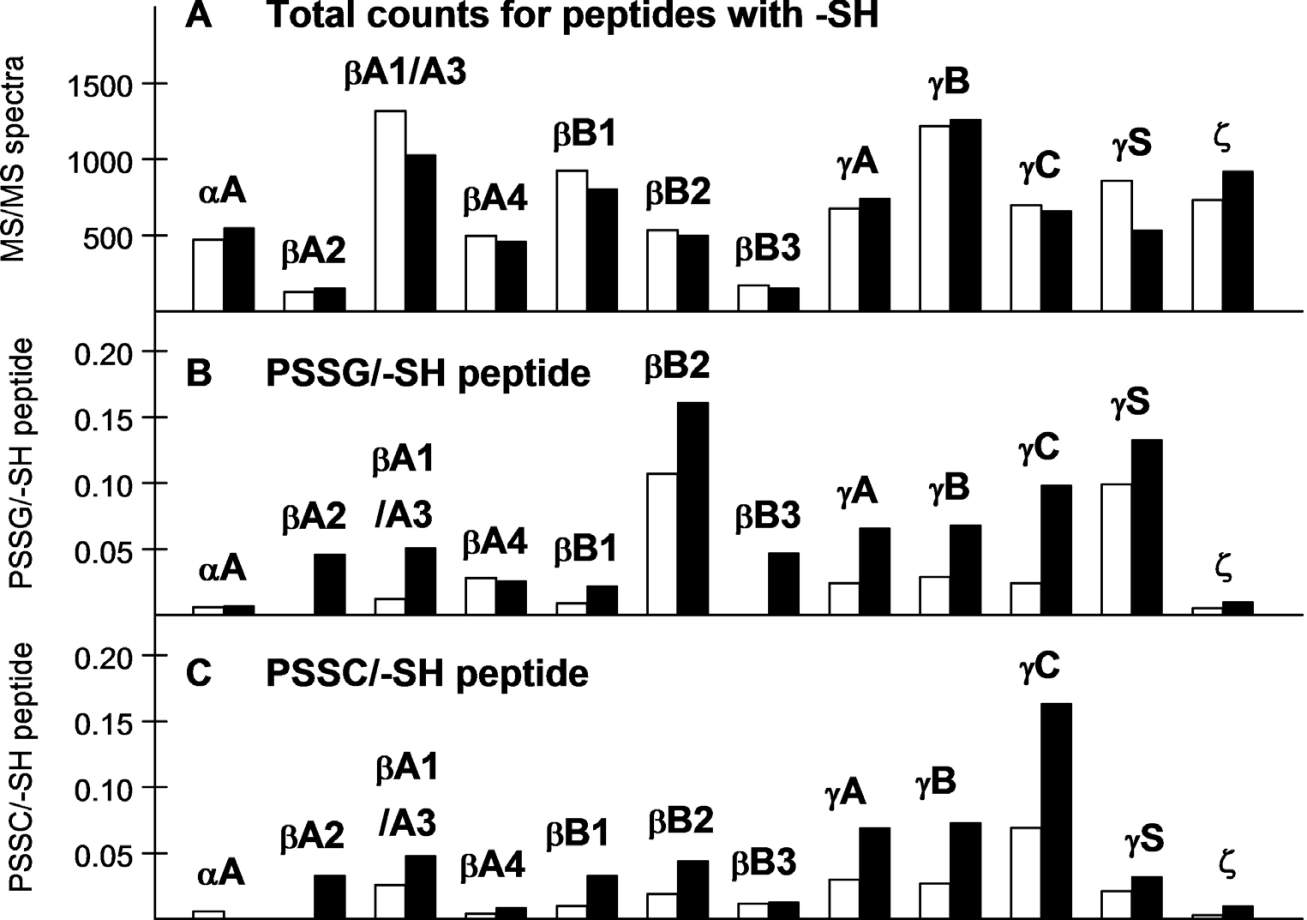
The numbers of tandem mass (MS/MS) spectra are shown for peptides of each guinea pig lens water-soluble nuclear crystallin. **A** shows total counts for peptides containing an -SH group; **B** shows peptides containing protein-bound glutathione (PSSG) expressed as per -SH peptide; and **C** shows peptides containing protein-bound cysteine (PSSC) expressed as per -SH peptide. Open bars are counts from age-matched controls and solid black bars are counts after 30 treatments of the animals with hyperbaric oxygen. There is a different vertical scale for **A**, compared to those for **B** and **C**, which are identical. Results for γN-crystallin are not shown because of a low number of detected total peptides (<50 counts). The counts in **A** correspond to a soluble protein sample of 0.4 mg. Seven crystallins (βA1/A3, βA4, βB1, βB2, βB3, γC, and γS) showed an O_2_-induced decrease in counts for peptides containing a cysteine residue, while 5 crystallins (αA, βA2, γA, γB and ζ) showed an increase (**A**). All the crystallins except αA and βA4 showed an O_2_-induced increase in PSSG level (**B**). βB2-crystallin exhibited the highest levels of control as well as O_2_-induced PSSG. In **C**, all the crystallins except αA and βB3 showed an O_2_-induced increase in PSSC level. γC-crystallin exhibited the highest levels of control as well as O_2_-induced PSSC.

The data were analyzed to determine which crystallin –SH peptides showed a loss as the result of O_2_ treatment (since the same amounts of protein were added for the control and experimental, a loss of one crystallin would necessarily be compensated for by an increase in another). Of the 12 guinea pig lens nuclear crystallins examined, seven (βA1/A3, βA4, βB1, βB2, βB3, γC, and γS) showed a relative decrease in soluble –SH peptides following HBO treatment, while five (αA, βA2, γA, γB, and ζ) exhibited an increase (Figure 1A). Major O_2_-induced changes in the levels of the –SH peptides included those for βA1/A3-crystallin (22% loss), βB1-crystallin (13% loss), βB2-crystallin (7% loss), γA-crystallin (10% gain), γS-crystallin (38% loss), and ζ-crystallin (26% gain; Figure 1A).

Figure 1B,C shows the effects of HBO treatment on the spectral counts for peptides containing either bound glutathione (B) or bound cysteine (C). The data are expressed as counts obtained for each mixed disulfide per the counts for total –SH peptides for each crystallin, which normalizes the data to account for differences in the abundance of the various crystallins, as well as any positive or negative changes in −SH peptide levels as a result of HBO treatment. Control counts for soluble nuclear PSSG (the open bars in Figure 1B) were relatively low for each crystallin except βB2 and γS, which were about seven times the average control value for the rest of the crystallins. All crystallins except βA4 showed marked increases in PSSG levels following HBO treatment (Figure 1B). The PSSG levels for βA2- and βB3-crystallins were undetectable for the controls, but increased substantially after HBO treatment. The total HBO-treated PSSG counts were twice those of the controls. The control counts for PSSC (the open bars in Figure 1C) were relatively low for each crystallin except γC. All crystallins, except αA and βB3, showed an increase in PSSC levels following HBO treatment (Figure 1C). O_2_-induced increases in PSSC levels were two- to threefold for most of the crystallins. The total HBO-treated PSSC counts were 2.1 times the control counts.

**Figure 2:**
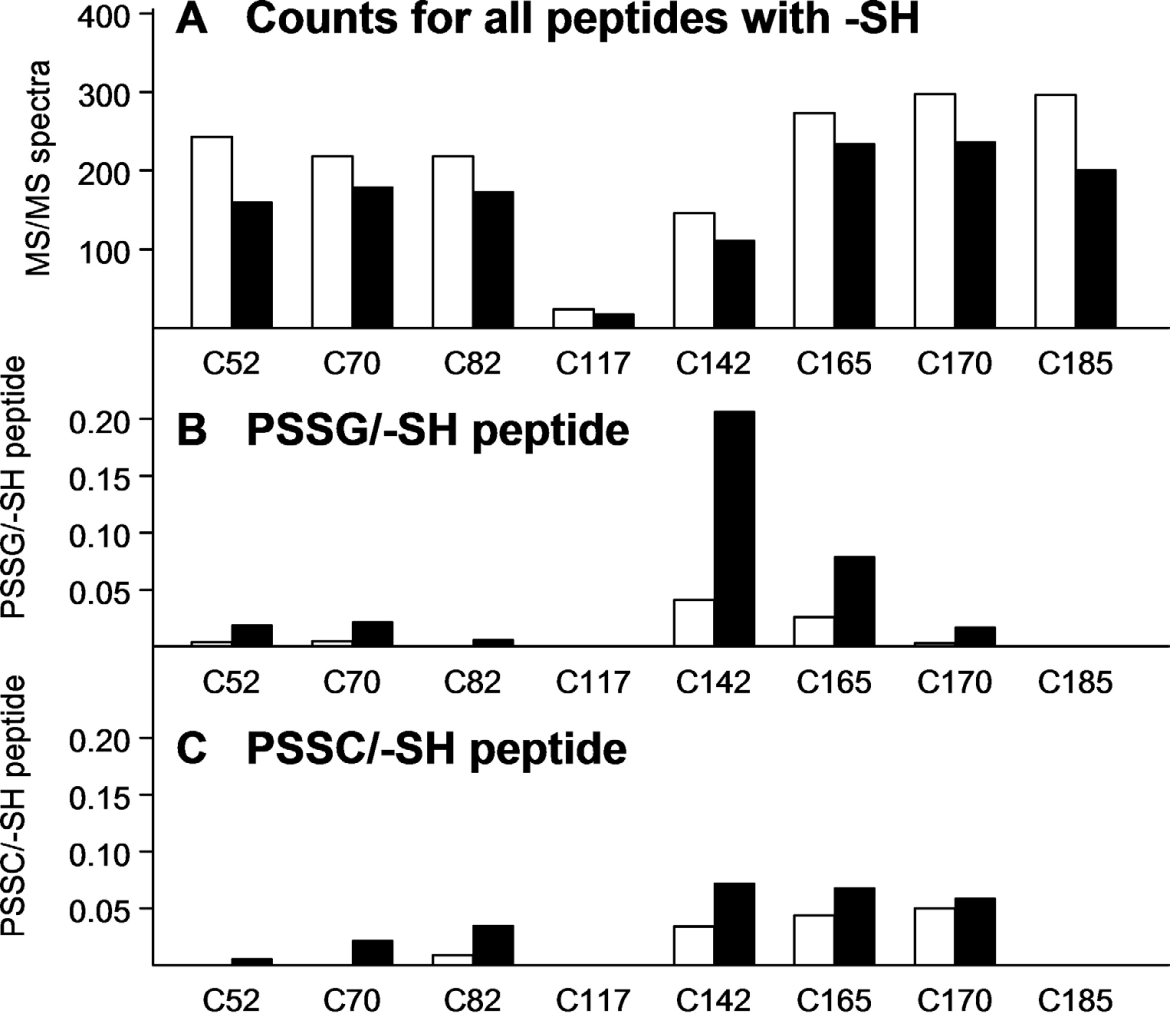
The numbers of tandem mass (MS/MS) spectra are shown for the 8 -SH group-containing peptides of water-soluble nuclear βA1/A3-crystallin. **A** shows total counts for each of the 8 peptides containing an -SH group; **B** shows peptides containing protein-bound glutathione (PSSG) expressed as per -SH peptide; and **C** shows peptides containing protein-bound cysteine (PSSC) expressed as per -SH peptide. Open bars are counts from age-matched controls and solid black bars are counts after 30 treatments of the animals with hyperbaric oxygen. There is a different vertical scale for **A**, compared to those for **B** and **C**, which are identical. The counts in **A** correspond to a soluble protein sample of 0.4 mg. Peptides for each of the 8 cysteine residues in panel **A** showed an O_2_-induced decrease in number of counts. A relatively low number of counts were detected for the C117 control and O_2_-treated peptides. The majority of O_2_-induced PSSG (**B**), and to a lesser extent PSSC (**C**), was shown by residues C142 and C165.

**Figure 3:**
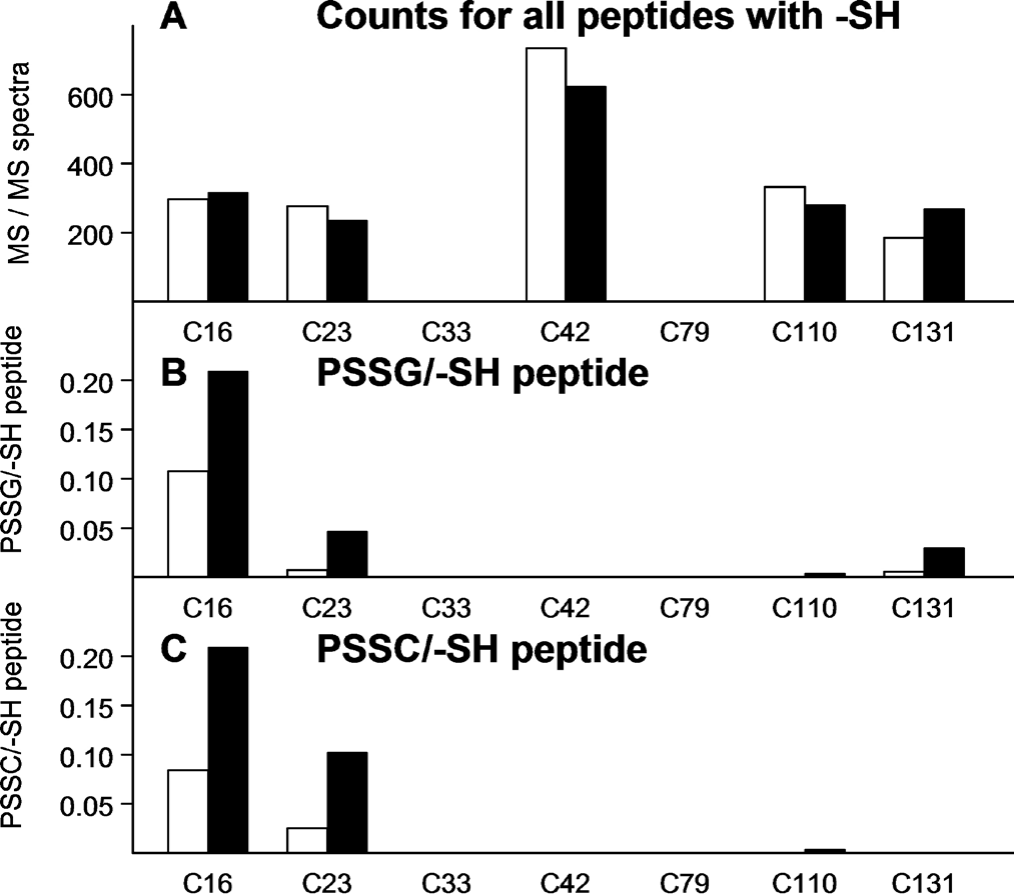
The numbers of tandem mass (MS/MS) spectra are shown for the 7 -SH group-containing peptides of water-soluble nuclear γB -crystallin. **A** shows total counts for each of the 7 peptides containing an −SH group; **B** shows peptides containing protein-bound glutathione (PSSG) expressed as per −SH peptide; and **C** shows peptides containing protein-bound cysteine (PSSC) expressed as per −SH peptide. Open bars are counts from age-matched controls and solid black bars are counts after 30 treatments of the animals with hyperbaric oxygen. There is a different vertical scale for **A**, compared to those for **B** and **C**, which are identical. The counts in panel **A** correspond to a soluble protein sample of 0.4 mg. Residue C42 showed a relatively high number of peptide counts for control and O_2_-treated samples due to the peptides containing C42 being identical in sequence for γA-, γB- and γC-crystallins (**A**). No detectable counts were obtained for residues C33 and C79 for either the control or O_2_-treated samples. Residues C16 and C23 accounted for the majority of O_2_-induced PSSG (**B**) and PSSC (**C**).

We also examined the number of –SH peptide counts recorded for each of the 61 cysteine residues of the 12 crystallins listed in Table 1. We found that 16 of the residues showed low or undetectable counts, including those for βA1/A3, C117 (Figure 2A); βA2, C13, C27, and C100 (not shown); βA4, C5 and C99 (not shown); βB3, C207 (not shown); γA, C33 (not shown); γB, C33 and C79 (Figure 3A); γC, C33 and C79 (not shown); γS, C37, C83, and C130 (not shown); and ζ, C239 (not shown). Tandem mass spectra for representative modified peptide forms for cysteine-containing peptides are shown in Appendix 2.

**Figure 4:**
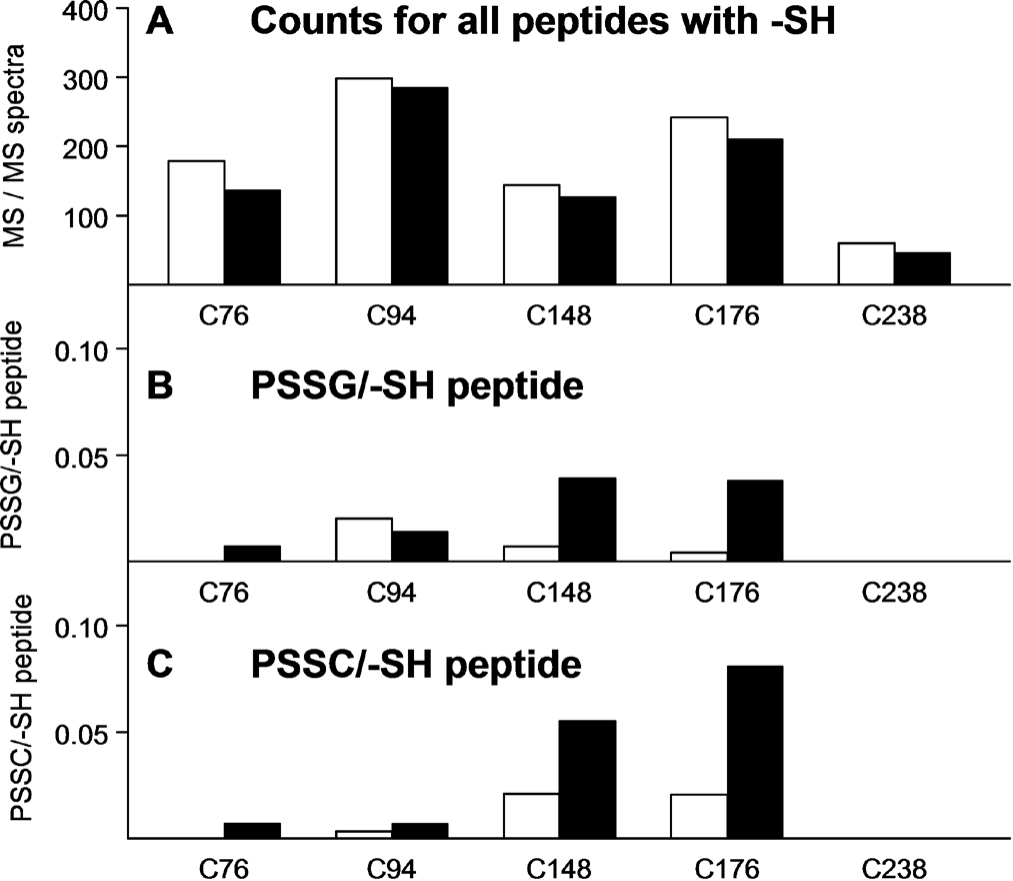
The numbers of tandem mass (MS/MS) spectra are shown for the 5 -SH group-containing peptides of guinea pig lens water-soluble nuclear βB1-crystallin. **A** shows total counts for each of the 5 peptides containing an -SH group; **B** shows peptides containing protein-bound glutathione (PSSG) expressed as per -SH peptide; and **C** shows peptides containing protein-bound cysteine (PSSC) expressed as per -SH peptide. Open bars are counts from age-matched controls and solid black bars are counts after 30 treatments of the animals with hyperbaric oxygen. There is a different vertical scale for **A**, compared to those for **B** and **C**, which are identical. The counts in **A** correspond to a soluble protein sample of 0.4 mg. Peptides for each of the 5 cysteine residues showed an O_2_-induced decrease in number of counts (**A**). The majority of O_2_-induced PSSG (**B**) and PSSC (**C**) were shown by residues C148 and C176.

**Figure 5:**
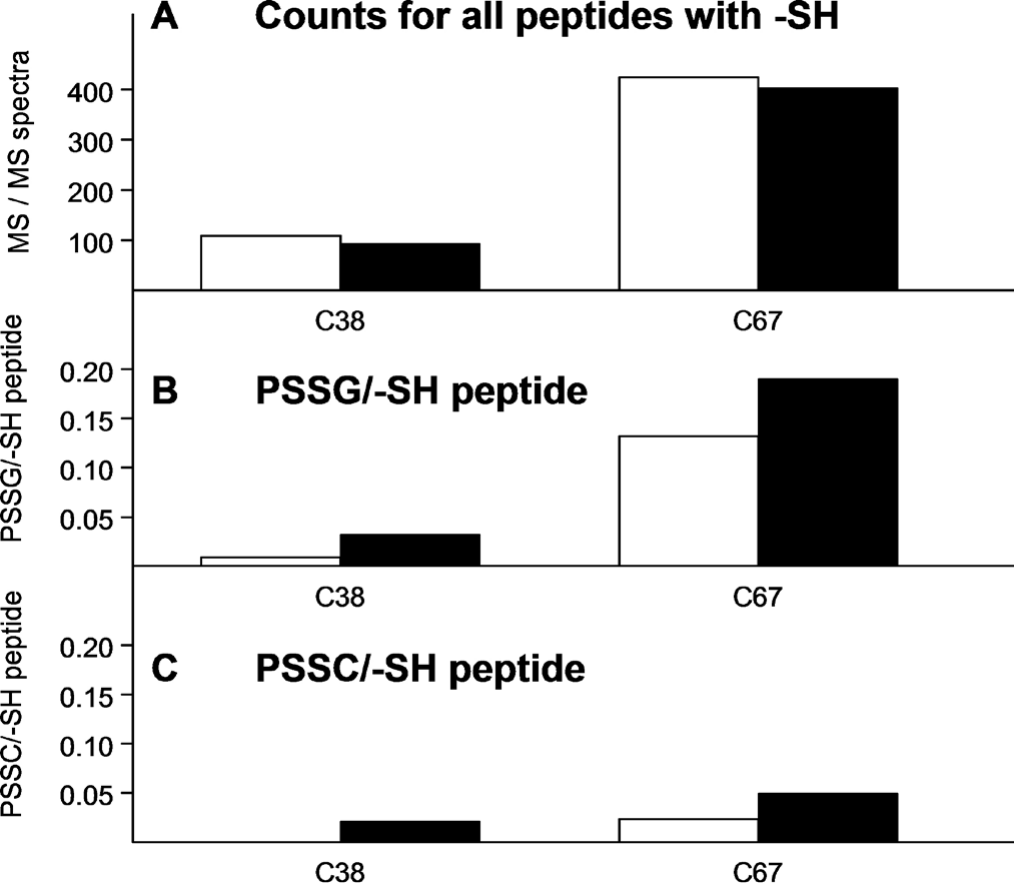
The numbers of tandem mass (MS/MS) spectra are shown for the 2 -SH group-containing peptides of water-soluble nuclear βB2-crystallin. **A** shows total counts for each of the 2 peptides containing an −SH group; **B** shows peptides containing protein-bound glutathione (PSSG) expressed as per −SH peptide; and **C** shows peptides containing protein-bound cysteine (PSSC) expressed as per −SH peptide. Open bars are counts from age-matched controls and solid black bars are counts after 30 treatments of the animals with hyperbaric oxygen. There is a different vertical scale for **A**, compared to those for **B** and **C**, which are identical. The counts in panel **A** correspond to a soluble protein sample of 0.4 mg. **A** shows that peptides for each of the 2 cysteine residues showed a slight O_2_-induced decrease in number of counts. The majority of control and O_2_-induced PSSG was shown by residue C67 (**B**). Note that this single residue of βB2 produced the highest levels of control and O_2_-induced PSSG, compared to the other 11 crystallins (see Figure 1B). Residue C67 produced relatively little control or O_2_-induced PSSC (**C**), compared to the amounts observed for PSSG (**B**).

We selected four crystallins, βA1/A3, γB, βB1, and βB2, to examine in more detail regarding the effects of O_2_ on modifying individual cysteine residues (Figure 2, Figure 3, Figure 4, and Figure 5, respectively). The control counts for the −SH peptides of βA1/A3-crystallin (the open bars in Figure 2A) were approximately equal for each of the eight cysteine residues except C117 and C142, which were lower. Following the HBO treatment, the counts decreased for all eight residues, compared to the control (Figure 2A). The control counts for PSSG, expressed per –SH peptide, were low for C52, C70, C82, C117, C170, and C185 (Figure 2B). Following HBO treatment, PSSG values increased for six of the eight cysteine residues, with more than 50% of the bound glutathione occurring at residue C142, fivefold higher than the control. Similar to the results for PSSG, HBO treatment also produced an increase in PSSC values for six of the eight cysteine residues; however, the increase in PSSC at residue C142 was only twofold over the control (Figure 2C). Residue C185 exhibited no control or O_2_-induced binding of either glutathione or cysteine (Figure 2B,C), despite showing substantial –SH peptide counts (Figure 2A).

For the –SH peptides of γB-crystallin, the control counts (the open bars in Figure 3A) were similar for the residues C16, C23, C110, and C131, but higher for C42, and zero for C33 and C79. The larger number of counts for C42 was due to tryptic peptides containing C42 being identical in sequence for the γA-, γB-, and γC-crystallins. O_2_ treatment produced a 15% loss of –SH peptides for residues C23, C42, and C110, with a 45% gain for C131 (Figure 3A). Residue C16 bound substantial amounts of glutathione and cysteine, in control as well as HBO-treated samples (Figure 3B,C). For PSSG, the C16 residue accounted for 90% of the total control counts and 72% of the total HBO-treated counts. For PSSC, C16 accounted for 77% of the total control counts and 66% of the total HBO-treated counts. Following HBO treatment, the PSSG values increased for residues C16, C23, and C131 (Figure 3B), and the PSSC values increased for C16 and C23 (Figure 3C). Residue C42 was unusual in that it showed high counts for cysteine-containing peptides (Figure 3A), but no counts for binding of either glutathione or cysteine in either the control or HBO-treated samples (Figure 3B,C).

Regarding βB1-crystallin, O_2_ treatment caused modest decreases in the –SH peptide spectral counts for each of the five cysteine residues (Figure 4A). Residues C148 and C176 accounted for the majority of the O_2_-induced binding of glutathione (79% of the total counts) and cysteine (91% of the total counts; the solid bars in Figure 4B,C). O_2_-induced PSSG formation was several-fold higher than the control for C148 and C176 (Figure 4B), and PSSC formation was higher for these two residues (Figure 4C). Although C94 showed the highest total counts for –SH peptides (Figure 4A), it exhibited relatively low count values for glutathione- and cysteine-modified peptides (Figure 4B,C).

As indicated above (Figure 1B), βB2-crystallin showed the highest levels of glutathione-modified peptides of all the crystallins, for the control and HBO-treated samples. Figure 5B shows that nearly 97% of the total PSSG counts for this crystallin were associated with just one of its two cysteine residues, C67. HBO treatment produced only a small increase in PSSG counts over control for this residue. In contrast to the high level of PSSG formation for residue C67 (control and HBO-treated samples), the overall level of PSSC formation was much lower (PSSC/peptide versus PSSG/peptide; Figure 5B,C). PSSC formation also strongly favored the C67 site, compared to C38.

Of the five cysteine residues of ζ-crystallin, only C166 showed a significant O_2_-induced increase in mixed disulfide formation (a threefold increase in PSSC); peptide counts for C239 were very low, compared to the counts for the other four residues. Of the seven cysteine residues of γS-crystallin, the peptide counts for C37, C83, and C130 were very low, compared to counts for the other four residues. The PSSG levels were high for C25 and C27, for the control and HBO-treated samples. Although the data for ζ- and γS-crystallins are not shown here, they appear in Appendix 5.

The last column in Table 1 indicates the number of cysteine residues for each guinea pig lens nuclear crystallin that were modified by glutathione or cysteine in either the control or HBO-treated samples. All major crystallins, except αB with no cysteines, were modified to some extent by S-glutathiolation or S-cysteinylation. Overall, 72% of the cysteine residues in the guinea pig lens nucleus were capable of binding glutathione, cysteine, or both molecules. The crystallin with the highest level of modification was βA1/A3 (six of eight –SH groups), and that with the lowest (two of five –SH groups) was βA2, perhaps due to its low abundance.

## Discussion

This study demonstrated the value of bottom-up proteomics for investigating binding of glutathione and cysteine to specific lens crystallin sites in an animal model for cataract. The technique's picomole sensitivity allowed use of only two guinea pig lens nuclei, having a total wet weight of 40 mg, for analysis of 12 water-soluble crystallins containing 61 individual cysteine residues. More than 70% of the sites were capable of binding glutathione, cysteine, or both molecules (Table 1). Although other studies have employed mass spectrometry to investigate oxidation of −SH groups in lens crystallins [32-35], none have had the ability to analyze so many crystallins and cysteine residues at one time.

Comparison with previous cDNA transcript and protein data demonstrates the reliability of the spectral counting method used in this study. The observed five most abundant crystallins in the 20-month-old guinea pig lens nucleus, ζ, γS, βB2, αA, and γB (Table 1, columns 2 and 3), were also the five most abundant cDNA transcripts reported previously for the young guinea pig lens [36]. ζ-crystallin, which showed 16% abundance in this study, has been reported to comprise about 10% of the total guinea pig lens protein [37,38] in the cortex and nucleus [36]. The abundance of γS-crystallin in the lens nucleus was surprising since this protein is reportedly expressed only after birth, with the protein’s synthesis increasing with age [39]. βB2-crystallin is the most abundant basic β-crystallin in the lens [40], and a major protein in the guinea pig lens nucleus [36]. In previous work, cDNA transcripts of αA-crystallin in the young guinea pig lens (2.5 months old) were reported to be more abundant than transcripts of ζ-crystallin, and two-dimensional electrophoretic gel analysis of 2.5-month-old guinea pig lens nuclear proteins showed the level of αA-crystallin protein was substantially higher than that for ζ-crystallin [36]. The lower abundance of αA-crystallin peptides in the current study may have been due in part to a greater loss of water-soluble αA-crystallin protein in the guinea pig lens nucleus, as the animal aged from 2.5 to 20 months. The twofold relative abundance of αA-crystallin peptides to those for αB in the 20-month-old lens nucleus (Table 1, column 2) differs from an 8:1 ratio of αA/αB protein in the young guinea pig lens nucleus [36]. Relative peptide counts for γA-, γB-, and γC-crystallins (Table 1, column 2) were similar to those reported previously for corresponding cDNA transcripts [36]. In contrast, levels of protein for the three γ-crystallins were shown to be about equal in the young guinea pig lens nucleus [36]. Again, aging may have contributed to the observed differences in the relative abundances of the γ-crystallins observed in the present study. The failure to observe any peptide counts for γD-, γE-, and γF-crystallins agrees with a previous report of an absence of cDNA and protein for these crystallins in the guinea pig lens [36]. γN-crystallin showed 100-fold lower levels of peptides (Table 1), as well as cDNA transcripts [36], compared to those for the most abundant crystallin.

The protocol used in this study, treatment of guinea pigs 30 times with HBO over a 2.5-month period, is known to produce a moderate increase in the level of lens nuclear light scatter [1,41], with five- to sixfold increases in the levels of lens nuclear PSSG and PSSC [1]. Thus, the study, while providing a valuable data set, represents only one point on the time course toward the development of nuclear cataract. The degree of nuclear light scatter increases substantially after 50, 65, and 80 HBO treatments of the animals [1,26,41], without additional increases in PSSG and PSSC [1]. At the 30-treatment period, the loss of WS protein in the lens nucleus is 16% [1]. In the current study, we showed that seven crystallins (βA1/A3, βA4, βB1, βB2, βB3, γC, and γS) exhibited a decrease in soluble nuclear −SH peptides following 30 HBO treatments (Figure 1A), presumably due to relatively greater O_2_-induced disulfide-crosslinking and precipitation of these crystallins, compared to the others. Several earlier studies also found that β-crystallins were more prone to insolubilization following exposure to oxidative stress, compared to other crystallins [42-44]. In a prior investigation where rabbit lenses were treated in vitro with HBO, β-crystallins were the first proteins to form high molecular weight proteins, presumably because of disulfide crosslinking, before becoming insoluble [45]. γS-crystallin, which exhibited the greatest O_2_-induced loss of any of the crystallins (Figure 1A), has been reported to undergo disulfide-crosslinking as a major post-translational modification in the aging human lens [46].

In contrast, the γA-, γB-, and γC-crystallins appeared to be less susceptible to O_2_-induced loss, compared to the β-crystallins (Figure 1A). Whereas the β-crystallins exhibited an overall 13% relative loss in soluble nuclear –SH peptides following O_2_ treatment, the γ-crystallins (A, B, and C) showed a slight overall increase (Figure 1A). The data, however, do not indicate a clear-cut protective effect of glutathiolation and/or cysteinylation in preventing insolubilization of the γ-crystallins. The overall increases in O_2_-induced binding of glutathione and cysteine to the γ-crystallins (A, B, and C) and β-crystallins were about the same, two- to threefold (Figure 1B,C). We know that after long-term treatment of guinea pigs with HBO (80 treatments, compared to the 30 of this study), β- and γ-crystallins become disulfide-crosslinked in the water-insoluble protein fraction [26]. Thus, PSSG and PSSC formation may act to delay O_2_-induced insolubilization of both types of crystallins, but with a greater effect on the γ-crystallins at an early stage of oxidative stress. This conclusion supports an earlier hypothesis that glutathiolation and cysteinylation of soluble proteins act to delay irreversible formation of protein disulfide [17], but this conclusion also supports the contention by Lou that PSSG and PSSC are precursors of protein–protein disulfide cross-links [47]. Glutathiolation can be reversed if a substantial reduced to oxidized glutathione ratio is restored [16], but under conditions of continued oxidative stress, PSSG and PSSC may become precursors of PSSP. Additional support for a protective role for PSSG comes from a study showing that glutathione-modified βB2-crystallin in normal old human lenses is present only in the soluble protein fraction and is more resistant to heat-induced precipitation; in the water-insoluble fraction, βB2 is modified entirely by disulfide-crosslinks [48], supporting an earlier contention that glutathione adducts increase lens crystallin solubility [49].

Effects of 30 HBO treatments on two major crystallins in the guinea pig lens, αA and ζ, were unremarkable regarding the loss of –SH peptides and the formation of mixed disulfide. Both proteins showed O_2_-induced increases in peptide levels, instead of the relative losses exhibited by the β- and γS-crystallins (Figure 1A), and both proteins also showed relatively low levels of PSSG and PSSC formation in control as well as O_2_-exposed samples (Figure 1B,C). Guinea pig αA-crystallin contains only one –SH group located at residue C131, compared to two –SH groups for human αA at C131 and C142. Glutathione adducts to C131 and C142 of human αA-crystallin have been detected in lenses of renal failure patients, but not in normal old human lenses [50], and in vitro binding of glutathione to the two –SH groups of human αA produced substantial loss of its chaperone-like activity [51]. Intramolecular disulfide-crosslinking of the two cysteine residues of αA-crystallin in young human lenses has been reported [52], and in old, normal human lenses, 77% of αA-crystallin present in the water-insoluble protein fraction was disulfide-crosslinked [18]. Of the five cysteine residues of ζ-crystallin, only C166 showed significant mixed disulfide formation (PSSC) after the 30 HBO treatments (data not shown). C248, which has been reported to be important for quinone oxidoreductase activity of the guinea pig protein [53], showed only minimal PSSG and PSSC formation. Why αA- and ζ-crystallin did not bind more glutathione and cysteine after 30 HBO treatments is not clear. The large size of each native protein (800 kDa for α-crystallin and 140 kDa for ζ-crystallin) may have been a contributing factor, and the binding of nicotinamide adenine dinucleotide phosphate to ζ-crystallin has been shown to offer some protection against sulfhydryl reagents and H_2_O_2_ [54]; an –SH group appears to be near the nicotinamide adenine dinucleotide phosphate binding site [55]. We know that after 80 treatments of guinea pigs with HBO, substantial amounts of disulfide-crosslinked αA- and ζ-crystallin are present in the water-insoluble (WI) fraction of the lens nucleus [26], and disulfide-crosslinked αA-crystallin exists to a large extent in mature human nuclear cataracts [9,34]; thus, the proteins eventually participate in crosslinking. Overall, the results suggest that the –SH groups of αA- and ζ-crystallin may be less prone to O_2_-induced oxidation compared to the other crystallins, but based on previous studies, the –SH groups of αA- and ζ-crystallin eventually do crosslink, resulting in protein precipitation; whether they form PSSG and/or PSSC before disulfide-crosslinking is not yet known.

Low or undetectable –SH peptide counts were recorded for 16 of the 61 cysteine residues, excluding those for γN-crystallin. Two of the residues were C33 and C79 of γB-crystallin (Figure 3A). These cysteines are found in predicted tryptic peptides of only five and three amino acids in length, respectively, which are too short to produce MS/MS data that can be matched to peptide sequences by SEQUEST. In a previous proteomic analysis of oxidation of crystallin cysteine residues in human lenses, the investigators were unable to obtain information on eight of 38 cysteine residues, four of which were also found to be undetectable in the current study: βA4 (C5) and γS (C37, C83, and C130) [34].

Of the eight cysteine residues of βA1/A3-crystallin, C142, and to a lesser extent, C165, accounted for the majority of O_2_-induced bound glutathione, but without the same high level of bound cysteine (Figure 2B,C). If the βA1/A3-crystallin dimer adopts a conformation similar to that of the βB2 dimer [56], the two C142 residues would be located near the connecting peptides close to four positively charged lysine residues. Negatively charged oxidized glutathione might be attracted to these positively charged residues, resulting in binding of glutathione to the C142 residue; neutral cystine would presumably not be attracted in the same way. Nearby positively charged amino acid residues have been linked previously with increasing the activity of cysteine residues [57]. It is surprising that C142 and C165 change to serines in human βA1/A3-crystallin, while being conserved in other species, including guinea pig, bovine, dog, frog, mouse, and rabbit [58]. How this might affect the response of human βA1/A3 to oxidative stress is unclear. Only slight O_2_-induced formation of PSSG and PSSC was observed for residues C52 and C170 of βA1/A3-crystallin (Figure 2B,C); however, extensive oxidation of these two residues has been observed in a proteomic analysis of human nuclear cataracts [34]. Despite the presence of abundant peptides for residue C185 of βA1/A3, we found no evidence for either PSSG or PSSC formation in either the control or O_2_-treated samples (Figure 2); however, intramolecular crosslinking of C185 with C170 has been reported for βA1/A3-crystallin present in human nuclear cataracts [59]. C82 and C117 were found to be glutathiolated even in newborn human lenses [35], but these two residues were not modified by glutathione to any extent in the guinea pig lens (Figure 2B).

Of the seven cysteine residues of guinea pig γB-crystallin, C16 and C23 accounted for nearly all of the control and O_2_-induced formation of PSSG and PSSC (Figure 3B,C, respectively). Similar results were observed for the γA- and γC-crystallins (data not shown). The three-dimensional structure of bovine γB-crystallin has been determined at high resolution [60,61]. Residue C16 of the calf crystallin has been shown to be by far the most exposed of that protein's seven –SH groups [61,62]. Solvent accessibility values for the three most exposed sulfhydryls of calf γB were 65, 18, and 12 Å for C16, C23, and C42, respectively [62]. C16 is present in γB-crystallin of guinea pig, bovine, dog, mouse, and rat, but not of chimpanzee, human, and monkey [58]. In contrast, C23 and C42 are conserved for γB of the eight species mentioned above. Slingsby and Miller [63] found that three of the seven cysteines of bovine γB-crystallin reacted avidly with glutathione; we also observed three glutathione-reactive residues (Figure 3B). Hanson et al. [32] reported two glutathione adducts per molecule of bovine γB-crystallin following treatment of intact bovine lenses with H_2_O_2_.

Regarding the human lens, γC-crystallin is in relatively high concentration, compared to γB [64,65]. In human nuclear cataracts, residues C23, C79, and C153 of γC-crystallin have been reported to be oxidized, in contrast to C42, which remained reduced [34]. We found no formation of PSSG or PSSC at residue C42 of any of the γA-, γB-, or γC-crystallins, despite detecting relatively large numbers of –SH peptides for this site (Figure 3 shows the result for γB-crystallin).

βB1-crystallin (Figure 4) is a major protein in the young human lens, comprising 9% of the total crystallins in a newborn lens [66]. Of the five –SH groups of guinea pig βB1-crystallin, residues C148 and C176 accounted for the majority of the bound glutathione and cysteine, and showed high levels of O_2_-induced binding of both compounds (Figure 4B,C). These two residues are also found in βB1-crystallins of bovine, dog, mouse, and zebrafish, but not chimpanzee, human, or monkey, where the cysteines changed to serines [58]. In this regard, human βB1 lens protein would have less protein sulfhydryl redox buffer capacity [5,12] compared to that of the guinea pig. Residue C76, which showed relatively little binding of either glutathione or cysteine (Figure 4B,C), is highly conserved for eight other species in addition to the guinea pig, including bovine, chicken, chimpanzee, dog, human, monkey, mouse, and zebrafish [58]. This cysteine accounts for the only –SH group of human βB1. In the crystal form of truncated human βB1-crystallin, the single cysteine was oxidized to a sulfinic acid, due presumably to the long time required for crystallization of the protein [67].

βB2-crystallin showed the highest amount of PSSG formation (Figure 1B), almost all of it due to residue C67 (Figure 5B). Why the C67 site is so attractive for S-glutathiolation is not clear. βB2 is the least thermodynamically stable of any of the βγ-crystallins and actively partners with other β-crystallins, helping to keep them in solution [68-70]. In silico modeling showed that the β-hairpin region, which contains the C67 residue, participates early in the unfolding process [71], suggesting that increased accessibility of the residue may explain its unusual attraction for glutathione. Another reason may be the presence of three positively charged amino acids (two lysines and an arginine) close to C67 in the X-ray structure of βB2; no such groups are located close to C38 of the protein [72]. The C67 site is highly conserved in the lens βB2-crystallin of many species, including chicken, bovine, dog, human, mouse, and zebrafish [58,73]. Since βB2 accounts for up to 24% of the total soluble protein in the young human lens [64], βB2 can be considered a major contributor to PSH/PSSG redox buffer capability [5,16] in this lens. In old, normal human lenses, βB2-crystallin exhibits nearly 90% intramolecular disulfide-bonding in the water-insoluble fraction [18], and this same type of bonding is found for this protein in mature human nuclear cataracts [74].

Overall, 32 of 44 modified guinea pig crystallin cysteine residues (Table 1, column 7) were homologous with the human. The residues include αA: C131; βA1/A3: C52, C82, and C170; βA2: C33 and C119; βA4: C5, C33, C99, and C151; βB1: C76; βB2: C38 and C67; βB3: C39 and C45; γA: C16, C23, C111, and C130; γB: C23, C79, and C110; γC: C23, C33, and C109; γS: C23, C25, C27, C37, and C115; and ζ: C45 and C166. Why the active S-thiolation site of guinea pig βB2-crystallin (C67) has been conserved in the human protein, while similar sites for βA1/A3 (C142 and C165), βB1 (C148 and C176), and γB (C16) have not is unclear at this time. Determining whether loss of those S-thiolation sites is potentially harmful or beneficial for human lens nuclear transparency will require further investigation.

In summary, we used large-scale mass spectrometry methods to identify lens crystallin cysteine residues that bound glutathione and/or cysteine under normal and oxidative stress conditions. Since only two lens nuclei were used for the analyses, the animals selected may have been atypical; measurements in the future with additional animals and time points, possibly using targeted mass spectrometric approaches, would be beneficial. More than 70% of the 61 cysteine residues of 12 guinea pig lens nuclear crystallins were modified by S-glutathiolation or S-cysteinylation, demonstrating the substantial PSH redox buffer capability present in the center of the guinea pig lens.

## Appendix 1. Supplemental Methods.

To access the data, click or select the words “Appendix 1.” This will initiate the download of a pdf file.

## Appendix 2. Supplementary MS/MS spectra.

To access the data, click or select the words “Appendix 2.” This will initiate the download of a pdf file.

## Appendix 3. Protein summary and Quantitative summary.

To access the data, click or select the words “Appendix 3.” This will initiate the download of an Excel (xls) file.

## Appendix 4. Control peptides and Hyperbaric oxygen peptides.

To access the data, click or select the words “Appendix 4.” This will initiate the download of an Excel (xls) file.

## Appendix 5. Counts by site and Modified peptides.

To access the data, click or select the words “Appendix 5.” This will initiate the download of an Excel (xls) file.
